# The Associations Among Physical Activity, Quality of Life, and Self-Concept in Children and Adolescents With Disabilities: A Moderated Mediation Model

**DOI:** 10.3389/fped.2022.947336

**Published:** 2022-07-11

**Authors:** Wen Yang, Jane Jie Yu, Stephen Heung-Sang Wong, Raymond Kim-Wai Sum, Ming Hui Li, Cindy Hui-Ping Sit

**Affiliations:** ^1^Department of Sports Science and Physical Education, The Chinese University of Hong Kong, Hong Kong, Hong Kong SAR, China; ^2^Department of Sport and Exercise Science, Zhejiang University, Hangzhou, China

**Keywords:** adolescent, children, disability, physical activity, quality of life, self-concept

## Abstract

**Objectives:**

This study aimed to examine a moderated mediation model of the associations among physical activity (PA), quality of life (QoL), and self-concept (SC) in children and adolescents with physical disabilities (PDs) and intellectual disabilities (IDs).

**Materials and Methods:**

A total of 288 children and adolescents with PDs and IDs, aged between 6 and 17 years, were recruited from 16 special schools in Hong Kong. PA, QoL, and SC were measured using the Physical Activity Questionnaire for Older Children (PAQ-C), Paediatric Quality of Life Inventory (PedsQL), and Physical Self-Description Questionnaire Short Form, respectively. Mediation (i.e., PA, QoL, and SC) and moderation analyses (i.e., age, sex, and parental education level) were conducted by structural equation modelling (SEM) using the M plus and PROCESS macro for SPSS.

**Results:**

Physical activity (PA) was positively associated with SC (PDs: β = 0.373; IDs: β = 0.343), and SC significantly mediated the associations between PA and QoL in children and adolescents with PDs (parent proxy-report QoL: β = 0.114; self-report QoL: β = 0.141) and IDs (self-report QoL: β = 0.204). The mediating effects of SC accounted for 16 and 15% of the total effects of PA on QoL (parent proxy-report and self-report) respectively, in participants with PDs, and 8 and 4%, respectively, in those with IDs. Males and participants with higher parental education levels reported stronger associations among PA, QoL, and SC than their counterparts.

**Conclusion:**

This study supports mediating and moderating effects on the associations among PA, QoL, and SC in children and adolescents with PDs and IDs. Future researchers should consider PA intervention in mental health services and provide tailor-made PA programmes based on personal and environmental factors for children and adolescents with disabilities.

## Introduction

A disability is any condition of the body or mind that includes impairments, activity limitations, and participation restrictions according to the International Classification of Functioning, Disability and Health for Children and Youth (ICF-CY) framework (1). Disability is an interaction between personal and environmental factors that prevent individuals from fully participating in daily life activities (2). Approximately 1.5 billion people live with disabilities globally, and children and adolescents with physical disabilities (PDs) and intellectual disabilities (IDs) face greater risks of mental health problems and socioeconomic disadvantages, as well as less sufficient disease prevention and health promotion services, than their peers with typical development (3, 4). Evidence indicates that physical activity (PA) is positively associated with the psychosocial wellbeing of children and adolescents with disabilities; however, most of them are not physically active (4, 5), which may be detrimental to their mental health (2).

As an essential indicator of mental health, quality of life (QoL) refers to life satisfaction, including physical, emotional, social, and school wellbeing (6). Self-concept (SC) is one of the core indicators of QoL and is positively associated with QoL in children and adolescents with PDs and IDs (7–9). SC was defined as the self-perception of confidence, worth, competence, and abilities (10). According to SC theory, self-perception influences mental health through self-consistency and individual response, and improved self-perception positively affects mental health (11). QoL is achieved by satisfying basic psychological needs such as self-acceptance (12). Children and adolescents with disabilities are found to have low levels of QoL and SC (6, 9). Previous studies reported that children and adolescents with PDs had lower SC than those with IDs (13); and that age, environment, and/or significant others were significantly associated with QoL (14, 15) and SC (10) in children with disabilities.

There has been increasing research attention on PA, QoL, and SC in children and adolescents with PDs and IDs. For example, PA was positively associated with physical and social QoL in children and adolescents with PDs (16); PA experience had positive correlations with QoL in children and youth athletes with PDs (17); children and adolescents with PDs and IDs who participated in sports reported stronger associations among PA, QoL, and SC than those who did not participate in sports (18, 19); positive correlations were found between active leisure activities and athletic competence, and between skill-based leisure activities and physical appearance in adolescents with PDs (20). Evidence indicates that participation in PA promotes QoL through the improvement of physical self-perceptions in esteem and appearance as underlying psychosocial mechanisms in youth (12); however, the mechanisms still remain unclear in children and adolescents with PDs and IDs.

Some research gaps remain to be addressed. First, disability types such as PDs and IDs are associated with individualised needs and different environments (21); however, to the best of our knowledge, only one study reported the psychosocial health difference between students with PDs and IDs (13), which may hinder the understanding and promotion of PA and mental health in children and adolescents with different disability types. Second, previous studies found positive associations between PA and mental health in children and adolescents with PDs and IDs (16–19, 22), and the psychosocial mechanisms suggested the mediating role of SC in the associations between PA and QoL in youth (12). However, none of them reported the mediating effects using the structural equation modelling (SEM) on the associations among PA, QoL, and SC in children and adolescents with PDs and IDs. Third, while personal and environmental factors, such as age, sex, and parental education level, are essential components in the ICF-CY framework (1) and significantly associated with QoL and SC in children and adolescents with disabilities (10, 14), the moderated mediation model of the associations among PA, QoL, and SC are under-explored in children and adolescents with PDs and IDs. Further, parent proxy-report QoL had low to moderate correlations with self-report QoL in adolescents with IDs (6); however, the predictive effects of parent proxy-report QoL on self-report QoL in children and adolescents with PDs and IDs remain unknown. Therefore, in the present study, we aimed to (1) compare the levels of PA, QoL, and SC between children and adolescents with PDs and IDs; (2) examine the moderated mediation model, including the mediating effects of SC and the moderating effects of personal and environmental factors, of the associations among PA, QoL, and SC in children and adolescents with PDs and IDs; and (3) examine the predictive effects of parent proxy-report QoL on self-report QoL in children and adolescents with PDs and IDs.

## Materials and Methods

### Participants

**FIGURE 1:**
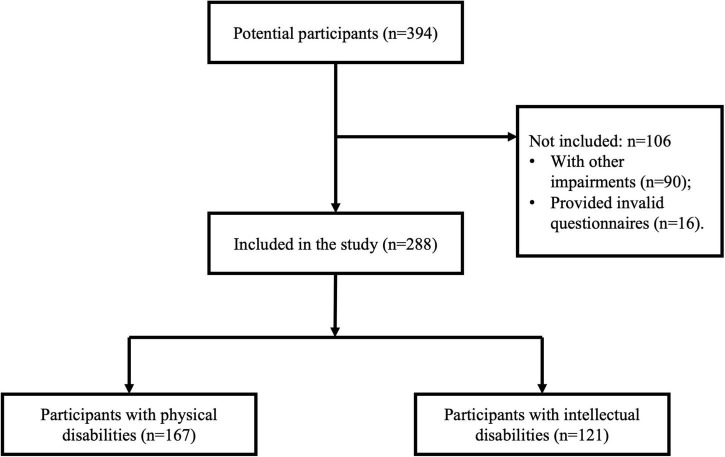
Strengthening the reporting of observational studies in epidemiology (STROBE) flowchart.

This cross-sectional study followed the Strengthening the Reporting of Observational Studies in Epidemiology (STROBE) checklist (23). Initially, we recruited 394 potentially eligible participants by sending invitations and interviewing teachers and parents in 16 special schools. The inclusion criteria of participants were (1) receiving education in special schools for students with PDs and IDs; (2) being able to understand the items in the questionnaires; and (3) being diagnosed with PDs by the Gross Motor Function Classification System (24), and mild to moderate IDs according to the Diagnostic and Statistical Manual of Mental Disorders, fifth edition (25); and (4) having no other impairments (e.g., visual or hearing impairment) or health conditions (e.g., asthma, cancer, and heart disease) that may limit PA participation. During the participants’ recruitment, 90 participants were excluded due to other impairments, and 16 participants provided invalid questionnaires in data collection. Finally, 288 children and adolescents with PDs and IDs aged between 6 and 17 years were included in the study (see Figure 1). Participants were recruited from 16 special schools, representing 32% of the 50 special schools for children and adolescents with PDs and IDs in Hong Kong. Two consent forms were obtained from participants and their parents, and all measures and procedures were approved by the Joint Chinese University of Hong Kong–New Territories East Cluster Clinical Research Ethics Committee (CREC Ref. No.: 2019.471).

### Measures

We assessed the PA levels of children and adolescents with disabilities using the Chinese version of the Physical Activity Questionnaire for Older Children [PAQ-C; (26)]. The PAQ-C evaluates the amount of moderate-to-vigorous PA (MVPA) over the last 7 days, and each item has five options, with higher scores indicating higher PA levels. The sample question in the physical education (PE) subscale was “in the last 7 days, during your PE classes, how often were you very active,” and the response options ranged from 0 (I do not do PE) to 5 (Always) (27). Consistent with previous studies (28, 29), the Cronbach’s alphas of the total PA and the subscales in the present study were satisfactory, ranging between 0.737 and 0.930 for participants with PDs, and 0.711 to 0.939 for those with IDs.

We used the Chinese version of the Paediatric Quality of Life Inventory (PedsQL), which evaluates physical, emotional, social, and school QoL in children and adolescents with disabilities, and psychosocial QoL is the average score of emotional, social, and school QoL (30). QoL in children and adolescents with disabilities has been assessed by parent proxy-report and children’s self-report, as both assessments provide a holistic picture of children’s function and activities, and their correlations are acceptable (6). The sample question of physical QoL in parent proxy-report was “a problem has your child had with: walking more than one block,” and self-report “how much of a problem has this been for you: it is hard for me to walk more than one block” (6). Each item on a 5-point Likert scale ranges from 0 (never) to 4 (almost always), and the scale was reverse-scored and linearly transformed to a 0–100 scale (0 = 100, 1 = 75, 2 = 50, 3 = 25, and 4 = 0); the higher the score, the greater the participants’ QoL (31). In the present study, the Cronbach’s alpha of the total QoL was satisfactory (parent proxy-report = 0.923; self-report = 0.902), with subscales ranging between 0.758 and 0.903 for participants with PDs. Similarly, the Cronbach’s alpha of the total QoL was deemed satisfactory (parent proxy-report = 0.897; self-report = 0.903), with subscales ranging between 0.740 and 0.912 for participants with IDs. The internal consistency was acceptable to good and consistent with previous studies (30, 31).

We employed the Chinese version of the Physical Self-Description Questionnaire Short Form (PSDQ-S) to test the SC of children and adolescents with disabilities (32). This test measures perceptions of PA, appearance, body fat, coordination, endurance, esteem, flexibility, global self, health, sport, and strength, and each subscale contains three to five items (33, 34). The sample question for coordination was “I feel confident when doing coordinated movement,” and each item had six options, ranging from 1 (False) to 6 (True) (34). Consistent with previous studies (33, 34), the Cronbach’s alpha for the total SC was acceptable to good for participants with PDs (0.917) and IDs (0.920). Meanwhile, the subscale of esteem was dropped for further analysis due to its low reliability (PDs = 0.529; IDs = 0.487). Other subscales showed acceptable to good internal consistency, ranging from 0.702 to 0.940 in participants with PDs and 0.726 to 0.913 in those with IDs.

### Procedure

Data were collected between January and June 2021. We visited the special schools and conducted data collection in the morning on weekdays based on the schedules of special schools. All questionnaires were interview-administered in the classroom, with the assistance of PE teachers, parents, and/or trained research assistants. Parents were also invited to provide demographic information about their children and complete the parent proxy-report QoL. The questionnaires took approximately 20–30 min to complete.

### Data Analysis

**FIGURE 2:**
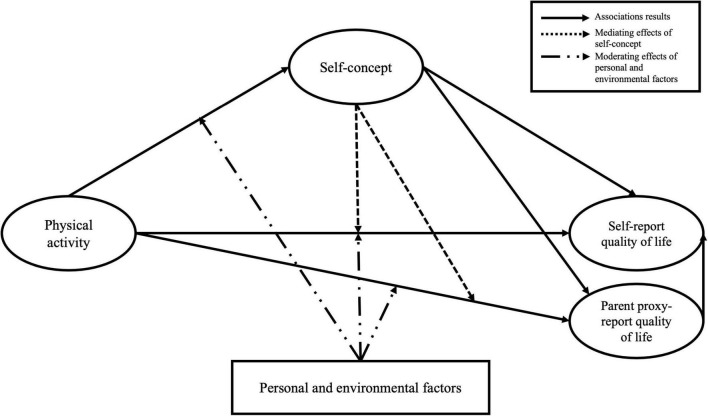
The hypothesised model among physical activity (PA), quality of life (QoL), and self-concept (SC) in children and adolescents with disabilities. Solid arrows denote association results; dotted arrows signal the mediating effects of SC on the associations between PA and QoL; dash-dotted arrows point to significant moderating effects of personal and environmental factors on the associations among PA, QoL, and SC.

We analysed data in a series of steps using IBM SPSS version 26.0 and M plus software version 8.3, and *p* < 0.05 was considered statistically significant. The first step of the analysis involved the use of descriptive statistics (means ± SDs or percentiles) to illustrate the characteristics of participants and variables. A multivariate analysis of variance (MANOVA) was used to compare the levels of PA, QoL, and SC between participants with PDs and IDs. The second step entailed presenting construct validity, including convergent validity (i.e., factor loading [≥0.5], average variance extracted [≥0.5], and composite reliability [≥0.7]) and discriminant validity (i.e., a correlation between latent variables being significantly smaller than 1), to assess the reliability and validity of the measurement model (35). The correlations among PA, QoL, and SC were calculated using Spearman rank correlation which indicates low (0.1), moderate (0.4), and strong (0.7) correlations (36). We used the SEM in step 3 to cross-validate the hypothesised model (see Figure 2). The mediating effects of SC on the associations between PA and QoL and the predictive effects of parent proxy-report QoL on self-report QoL were examined using 5,000 bootstrap samples, as 5,000 bootstrap samples are frequently used (37). A bias-corrected and accelerated 95% confidence interval (CI) and a maximum likelihood estimation method for missing data were used in the data analysis (38). The moderating effects of personal (i.e., age and sex) and environmental factors (i.e., parental education level) on the associations among PA, QoL, and SC were examined using SEM (39). We employed a combination of methods to assess model fit, which included (a) significant parameter estimates, (b) goodness of fit, (c) comparative fit index (CFI), (d) Tucker-Lewis index (TLI), (e) the standardised root mean square residual (SRMR), and (f) the root mean square error of approximation (RMSEA) (40). The rule of a reasonable cut-off for the fit index of CFI and TLI is 0.90; an SRMR value of less than 0.08 is considered a good fit, and less than 0.10 is acceptable; RMSEA values are often interpreted as follows: 0, a perfect fit; <0.05, a close fit; 0.05–0.08, a fair fit; 0.08–0.10, a mediocre fit; and >0.10, a poor fit (40). The fourth step of the analysis was to examine the mediating and moderating effects on the associations among total PA, QoL, and SC using SPSS PROCESS macro version 3.4.1 (41), with 5,000 bootstrap samples and a bias-corrected and accelerated 95% CI (42). The magnitude of the mediating effects was measured by R squared (*R*^2^), and the mediation and moderation analyses in PROCESS macro were adjusted by age, sex, and parental education level. PA was treated as the independent variable, with parent proxy-report and self-report QoL as dependent variables, SC as the mediator, and personal and environmental factors as moderators.

## Results

Table 1 presents the characteristics of children and adolescents with PDs and IDs, and 167 participants living with PDs (58%) and 121 with IDs (42%), with 194 boys (67.4%) and 94 girls (32.6%). The mean age was 11.63 ± 3.32 years. Regarding the parental education level, 163 parents (56.6%) had less than college/university education, and 121 parents (43.4%) had college/university education or higher.

**TABLE 1:** Characteristics of children and adolescents with disabilities (*N* = 288).

Characteristics	Dimension	Entire sample	Physical disability	Intellectual disability
Number	Overall	288	167 (58%)	121 (42%)
Age (years)	Overall	11.63 ± 3.32	11.63 ± 3.36	11.63 ± 3.27
Sex	Male	194 (67.4%)	101 (60.5%)	93 (76.9%)
	Female	94 (32.6%)	66 (39.5%)	28 (23.1%)
Parental education level	<College/University	163 (56.6%)	93 (55.7%)	70 (57.9%)
	≥College/University	125 (43.4%)	74 (44.3%)	51 (42.1%)
				

The descriptive statistics and missing data of PA, QoL, and SC in children and adolescents with PDs and IDs are shown in Table 2. There were significant differences between participants with PDs and IDs in PA (*F* = 4.95, *p* < 0.01), QoL (parent proxy-report: *F* = 29.12, *p* < 0.01; self-report: *F* = 23.67, *p* < 0.01), and SC (*F* = 9.41, *p* < 0.01), which illustrated higher levels of PA (2.00 ± 0.61), QoL (parent proxy-report: 63.82 ± 14.46; self-report: 62.71 ± 15.76), and SC (3.66 ± 0.72) in children and adolescents with IDs than those with PDs (PA: 1.71 ± 0.56; parent proxy-report QoL: 55.27 ± 18.87; self-report QoL: 56.56 ± 19.29; SC: 3.23 ± 0.86). Further, the correlations among PA, QoL, and SC in children and adolescents with PDs and IDs were low to moderate (see Table 3).

**TABLE 2:** Descriptive statistics of physical activity (PA), quality of life (QoL), and self-concept (SC) in children and adolescents with disabilities.

Variables	Physical disability	Intellectual disability	MANOVA	Missing data *N* (%)
**Physical activity (PA; range 1–5)**				
Checklist	1.30 ± 0.44	1.55 ± 0.54		4 (1.39%)
Physical education	2.58 ± 1.02	2.86 ± 0.96		2 (0.69%)
Recess	1.32 ± 0.68	1.80 ± 0.98		8 (2.78%)
Lunch	1.98 ± 1.25	1.98 ± 1.18		0 (0%)
After school	1.59 ± 1.02	1.98 ± 1.15		0 (0%)
Evenings	1.98 ± 1.15	2.19 ± 1.06		0 (0%)
Weekends	1.76 ± 1.07	2.10 ± 1.08		7 (2.43%)
Statement	1.96 ± 0.92	2.22 ± 0.89		2 (0.69%)
Weekly	2.01 ± 0.85	2.30 ± 0.85		5 (1.74%)
Total PA	1.71 ± 0.56	2.00 ± 0.61	*F*_(9_, _256)_ = 4.95**	22 (7.64%)
**Parent proxy-report quality of life (QoL; range 0–100)**				
Physical	51.64 ± 26.13	72.75 ± 17.81		6 (2.08%)
Emotional	69.88 ± 17.74	65.47 ± 17.14		3 (1.04%)
Social	51.86 ± 21.52	42.31 ± 23.68		2 (0.69%)
School	55.09 ± 18.07	58.37 ± 17.38		2 (0.69%)
Psychosocial	58.94 ± 16.06	55.10 ± 15.50		5 (1.74%)
Total parent proxy-report QoL	55.27 ± 18.87	63.82 ± 14.46	*F*_(4_, _273)_ = 29.12**	10 (3.47%)
**Self-report quality of life (QoL; range 0–100)**				
Physical	52.26 ± 25.99	70.19 ± 16.08		8 (2.78%)
Emotional	68.09 ± 18.21	66.80 ± 16.02		12 (4.17%)
Social	56.63 ± 22.95	49.34 ± 27.12		11 (3.82%)
School	55.00 ± 20.41	60.90 ± 17.44		8 (2.78%)
Psychosocial	60.62 ± 18.44	55.16 ± 21.11		12 (4.17%)
Total self-report QoL	56.56 ± 19.29	62.71 ± 15.76	*F*_(4_, _271)_ = 23.67**	12 (4.17%)
**Self-concept (SC; range 1–6)**				
Physical activity	2.78 ± 1.44	3.52 ± 1.20		5 (1.74%)
Appearance	4.03 ± 1.53	4.33 ± 1.29		3 (1.04%)
Body fat	3.50 ± 2.05	3.46 ± 1.77		2 (0.69%)
Coordination	3.08 ± 1.26	3.69 ± 1.10		7 (2.43%)
Endurance	2.42 ± 1.32	3.64 ± 1.16		3 (1.04%)
Flexibility	3.21 ± 1.32	3.97 ± 1.17		4 (1.39%)
Global self	3.23 ± 1.58	4.11 ± 1.21		3 (1.04%)
Health	3.62 ± 1.41	2.81 ± 1.35		4 (1.39%)
Sport	2.66 ± 1.58	3.70 ± 1.31		4 (1.39%)
Strength	3.50 ± 1.25	4.31 ± 1.14		3 (1.04%)
Total SC	3.23 ± 0.86	3.66 ± 0.72	*F*_(10_, _261)_ = 9.41**	16 (5.56%)

MANOVA, multivariate analysis of variance; **p < 0.01.

**TABLE 3:** The correlations among physical activity (PA), quality of life (QoL), and self-concept (SC) in children and adolescents with disabilities.

Variables	(1)	(2)	(3)	(4)
**Physical disability**				
(1) Physical activity	–	0.293**	0.197*	0.376**
(2) Parent proxy-report QoL	–	–	0.624**	0.397**
(3) Self-report QoL	–	–	–	0.556**
(4) Self-concept	–	–	–	–
**Intellectual disability**				
(1) Physical activity	–	0.163	0.124	0.427**
(2) Parent proxy-report QoL	–	–	0.621**	0.420**
(3) Self-report QoL	–	–	–	0.544**
(4) Self-concept	–	–	–	–

QoL, quality of life; *p < 0.05; **p < 0.01.

**FIGURE 3:**
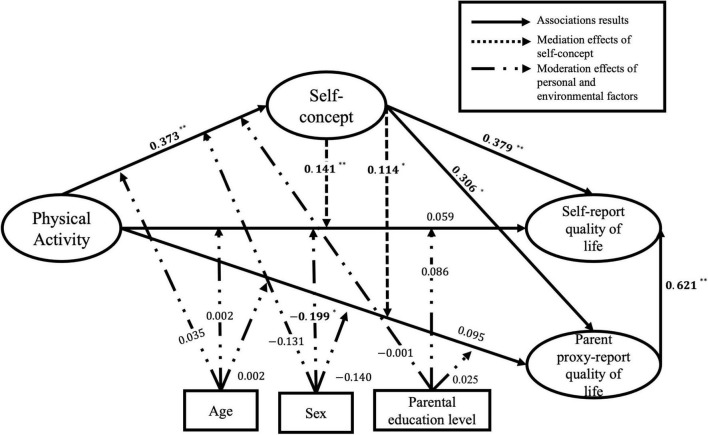
Standardised estimated coefficients of the structural equation modelling (SEM) in children and adolescents with physical disabilities (PDs). χ^2^_(175)_ = 328.084, *p* < 0.01, CFI = 0.937, TLI = 0.925, RMSEA = 0.074, SRMR = 0.075; bold are statistically significant; *p < 0.05; **p < 0.01.

**FIGURE 4:**
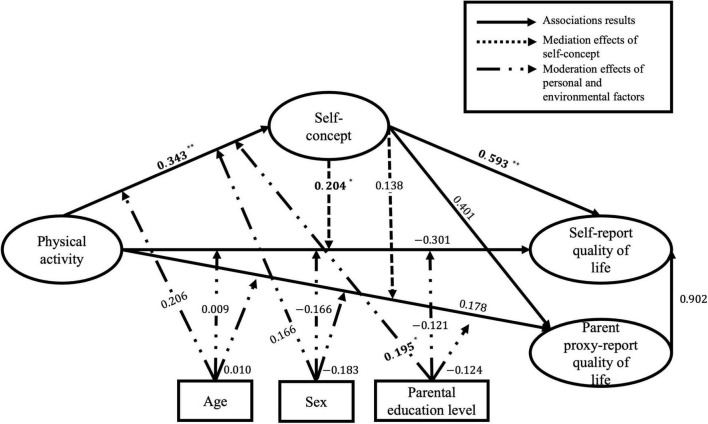
Standardised estimated coefficients of the structural equation modelling (SEM) in children and adolescents with intellectual disabilities (IDs). χ^2^_(175)_ = 294.879, *p* < 0.01, CFI = 0.918, TLI = 0.902, RMSEA = 0.085, SRMR = 0.073; bold are statistically significant; *p < 0.05; **p < 0.01.

The measurement model in participants with PDs and IDs showed acceptable validity and reliability with standardised factor loadings no less than 0.5, average variance extracted no less than 0.5, and composite reliability greater than 0.7. The SEM was tested to specify the structural model in children and adolescents with PDs and IDs based on 21 indicators (five for PA, eight for SC, and four each for parent proxy-report QoL and self-report QoL). The structural model for participants with PDs (see Figure 3) showed acceptable model fit indices: χ^2^_(175)_ = 328.084, *p* < 0.01, CFI = 0.937, TLI = 0.925, RMSEA = 0.074, SRMR = 0.075. Results showed that PA was positively associated with SC (β = 0.373, *p* < 0.01, 95% CI [0.210, 0.521]), and SC fully mediated the associations of PA with parent proxy-report (β = 0.114, *p* < 0.05, 95% CI [0.026, 0.266]) and self-report QoL (β = 0.141, *p* < 0.01, 95% CI [0.056, 0.256]), and the parent proxy-report QoL positively predicted self-report QoL in children and adolescents with PDs (β = 0.621, *p* < 0.01, 95% CI [0.377, 0.860]). Moreover, sex had a moderating effect on the association between PA and self-report QoL (β = −0.199, *p* < 0.05), which illustrated a stronger association between PA and self-report QoL in male participants than in their female counterparts. Further, the structural model for participants with IDs (see Figure 4) had acceptable model fit indices: χ^2^_(175)_ = 294.879, *p* < 0.01, CFI = 0.918, TLI = 0.902, RMSEA = 0.085, SRMR = 0.073. Results indicated that PA was positively associated with SC (β = 0.343, *p* < 0.01, 95% CI [0.087, 0.531]), and SC has been posited to fully mediate the association between PA and self-report QoL (β = 0.204, *p* < 0.05, 95% CI [0.035, 0.434]). There were no significant associations between parent-proxy report QoL and self-report QoL in children and adolescents with IDs (*p* > 0.05). Parental education level had positive moderating effects on the association between PA and SC (β = 0.195, *p* < 0.05), suggesting a stronger association between PA and SC in participants with higher parental education levels than in their counterparts.

PROCESS model 4 was utilised to investigate the mediating effects of total SC on the associations between total PA and SC, with model 59 for the moderating effects on the associations among total PA, QoL, and SC in children and adolescents with PDs and IDs, respectively. Results of mediation analysis (see Table 4) showed that total SC had significant mediating effects on the associations between total PA and QoL in children and adolescents with PDs (parent proxy-report QoL: β = 0.135, 95% CI [0.069, 0.217]; self-report QoL: β = 0.203, 95% CI [0.134, 0.281]) and IDs (parent proxy-report QoL: β = 0.206, 95% CI [0.055, 0.410]; self-report QoL: β = 0.305, 95% CI [0.114, 0.564]). The mediating effects of SC accounted for 16% (parent proxy-report QoL) and 15% (self-report QoL) of the total effects of PA on QoL in participants with PDs, and 8% (parent proxy-report QoL) and 4% (self-report QoL) in participants with IDs.

**TABLE 4:** Mediating effects of self-concept on the associations between physical activity and quality of life.

Dimension	PA-SC β	SC-QoL β	PA-QoL β	PA-SC-QoL			
				β	SE	95% CI	*R* ^2^
**Physical disability**							
Parent proxy-report QoL	0.391*	0.346**	0.178*	0.135	0.038	[0.069, 0.217]	16%
Self-report QoL	0.412**	0.492**	0.079	0.203	0.038	[0.134, 0.281]	15%
**Intellectual disability**							
Parent proxy-report QoL	0.444**	0.465**	0.012	0.206	0.093	[0.055, 0.410]	8%
Self-report QoL	0.456**	0.668**	−0.146	0.305	0.115	[0.114, 0.564]	4%

Standardised coefficient was used, and mediation analysis was adjusted by age, sex, and parental educational level; CI, confidence interval; PA, physical activity; QoL, quality of life; SC, self-concept; SE, standard error; *p < 0.05; **p < 0.01.

Results of the moderation analysis are presented in Table 5. The interaction between total PA and sex was negatively associated with total QoL in children and adolescents with PDs (parent proxy-report QoL: β = −16.248, *p* < 0.01; self-report QoL: β = −11.819, *p* < 0.05), which illustrated stronger associations between total PA and QoL in male participants than in their female counterparts.

**TABLE 5:** Moderating effects of personal and environmental factors on the associations among physical activity (PA), quality of life (QoL), and self-concept (SC).

Dimension	Age*Physical activity (PA) β	Sex*PA β	Parental education level *PA β
**Physical disability**			
Parent proxy-report QoL	–0.008	−16.248**	0.529
Self-report QoL	1.439	−11.819*	–3.022
Self-concept	0.001	0.007	–0.275
**Intellectual disability**			
Parent proxy-report QoL	0.623	–3.525	5.119
Self-report QoL	0.411	4.498	–9.630
Self-concept	0.036	–0.187	0.182

Unstandardised coefficient was used, and moderation analysis was adjusted by age, sex, and parental education level; QoL, quality of life; *p < 0.05; **p < 0.01.

## Discussion

This study examined a moderated mediation model of the associations among PA, QoL, and SC in children and adolescents with PDs and IDs. As hypothesised, we found positive associations between PA and SC and observed that SC significantly mediated the associations between PA and QoL in children and adolescents with PDs and IDs. Further, males and participants with higher parental education levels had stronger associations among PA, QoL, and SC than their counterparts.

In this study, PA was positively associated with SC in children and adolescents with PDs and IDs. Previous studies found that PA was positively associated with competence SC in children and adolescents with PDs (18); leisure activity was positively correlated with self-perceptions of appearance and competence in adolescents with PDs (20). Another study reported that PA might influence brain function, increase brain-derived neurotrophic factor, and promote social inclusion in children and adolescents (43). Biddle et al. suggested that psychosocial variables such as self-competence and confidence might change after PA participation, which could in turn positively affect mental health (44). However, the PA-SC association has been under-explored in children and adolescents with IDs, and more studies are needed to explore self-perception in this vulnerable group. Our study also found that SC significantly mediated the associations between PA and QoL in children and adolescents with PDs and IDs, consistent with previous studies. Evidence shows that SC is positively associated with QoL in children with PDs and IDs (7, 9); improved self-perception positively affects mental health through self-consistency and individual response according to the SC theory (11). Saha et al. (8) suggested that SC was negatively influenced by internalised stigma in children with disabilities, and that internalised stigma may impair their QoL (8). Psychosocial mechanisms recognised that SC mediates the associations between PA and QoL in youth without disabilities (12); however, the mediating effects of SC have rarely been investigated in children and adolescents with disabilities. Therefore, more studies are needed to investigate the mediating role of SC when planning PA programmes and mental health services in children and adolescents with disabilities.

In the present study, participants with IDs reported higher levels of PA, QoL, and SC than those with PDs. A previous study found that adolescents with IDs had better psychosocial health than those with PDs (13). The differences between participants with PDs and IDs may be due to inadequate persistence in task completion, under-developed ego orientation, and a lower ability to distinguish the factors leading to success or failure in adolescents with PDs (13). However, few studies have investigated the difference in PA levels between participants with PDs and IDs. Only one study compared MVPA levels across disability types, illustrating that boys with mild to moderate IDs and social developmental problems were more active than boys with severe ID (45). Thus, there is a need to develop quality PA measures and individualised PA programmes for children and adolescents with different disability types. Further, parent proxy-report QoL did not significantly predict the self-report QoL of participants with IDs in this study. A previous study found that parents reported lower QoL for their children than their children’s self-report (6). Future research is warranted to improve the agreement between parent proxy-report and self-report QoL in children and adolescents with IDs (6).

The present study found that sex and parental education level significantly moderated the associations among PA, QoL, and SC in participants with PDs and IDs, with stronger associations in males and participants with higher parental education levels than in their counterparts. A previous study found that sex significantly moderated the associations between body impairments and activity limitations according to the ICF-CY framework, with males having lower associations than females (46). Meanwhile, male participants reported greater brain-derived neurotrophic factor increases after exercise compared with their female counterparts (47), and boys with disabilities had higher PA levels than their girl counterparts (4). Previous studies also reported the moderating effects of parental education level. For example, as a critical indicator of socioeconomic and environmental factors, parental education level was significantly associated with PA levels in children with disabilities (48). Families with higher socioeconomic status had higher PA levels than families with lower socioeconomic status, and children with higher parental education levels had lower risks of mental disorders than those with lower parental education levels (15). As well, family relationship is a significant moderator in the associations between disability and activity limitations, which could be related to more social support from family members (46). However, we did not examine any family relationship in this study. Future research should explore the moderating effects of family relationship on the associations between PA and mental health in children and adolescents with disabilities. Furthermore, age had no moderating effects in this study. Only the chronological age was included in this study, and mental age should be considered in future studies.

This study has implications for PA participation and mental health in children and adolescents with disabilities. The findings suggested that PA might be the reason for mental health differences between participants with PDs and IDs, and PA has significant predictive effects on the formation of positive SC. Therefore, researchers and allied health care practitioners should consider PA intervention in mental health services for children and adolescents with PDs and IDs, and provide professional support to these two groups of children and their parents. Furthermore, the distinct levels of PA, QoL, and SC indicate different health conditions, functions, and needs of children and adolescents with PDs and IDs. Teachers may provide individualised PA programmes based on personal and environmental factors and improve the subjective experience of “being involved” for children and adolescents with disabilities (49). Parents may provide more assistance and support to promote PA levels and mental health for children and adolescents with PDs and IDs in the home environment, such as adopting active transportation, providing encouragement and company during PA participation, and communicating more with their children (50).

This study has several strengths. First, this was the first study to compare the levels of PA, QoL, and SC in children and adolescents with PDs and IDs, which could provide more health promotion information to meet the individual needs of children and adolescents with different disability types. Second, we examined the mediating (i.e., PA, QoL, and SC) and moderating effects (i.e., personal and environmental factors) by taking the psychosocial mechanisms, SC theory, and ICF-CY as theoretical frameworks into consideration. Third, this line of research was timely given the impacts of the COVID-19 pandemic on PA levels and mental health. Nevertheless, some limitations should be noted. First, we used subjective PA measures that may be subject to recall bias, and objective PA measures such as accelerometers are required to report PA levels. Second, we only considered two disability types comprising PDs and mild to moderate IDs, and the findings of our study cannot be generalised to other disability types including children and adolescents with severe ID. Finally, this study did not examine the causal relationship among PA, QoL, and SC in children and adolescents with disabilities, and future intervention studies are warranted.

## Conclusion

The current study suggested that PA was positively associated with SC in children and adolescents with PDs and IDs. SC significantly mediated the associations between PA and QoL based on psychosocial mechanisms and SC theory. Males and participants with higher parental education levels had stronger associations among PA, QoL, and SC than their counterparts, grounded in the ICF-CY framework. Future studies should consider PA in mental health services and provide individualised PA programmes for children and adolescents with PDs and IDs.

## Data Availability Statement

The original contributions presented in this study are included in the article/Supplementary Material, further inquiries can be directed to the corresponding author.

## Ethics Statement

The studies involving human participants were reviewed and approved by the Joint Chinese University of Hong Kong–New Territories East Cluster Clinical Research Ethics Committee. Written informed consent to participate in this study was provided by the participants or their legal guardian/next of kin.

## Author Contributions

WY: conceptualisation, investigation, and writing – original draft. JY, SW, RS, and ML: writing – review and editing. CS: conceptualisation, writing – review and editing, and supervision. All authors contributed to the article and approved the submitted version.

## Conflict of Interest

The authors declare that the research was conducted in the absence of any commercial or financial relationships that could be construed as a potential conflict of interest.

## Publisher’s Note

All claims expressed in this article are solely those of the authors and do not necessarily represent those of their affiliated organizations, or those of the publisher, the editors and the reviewers. Any product that may be evaluated in this article, or claim that may be made by its manufacturer, is not guaranteed or endorsed by the publisher.
